# Usage of Complementary Medicine in Switzerland: Results of the Swiss Health Survey 2012 and Development Since 2007

**DOI:** 10.1371/journal.pone.0141985

**Published:** 2015-10-29

**Authors:** Sabine D. Klein, Loredana Torchetti, Martin Frei-Erb, Ursula Wolf

**Affiliations:** Institute of Complementary Medicine, University of Bern, Bern, Switzerland; Sudbury Regional Hospital, CANADA

## Abstract

**Background:**

Complementary medicine (CM) is popular in Switzerland. Several CM methods (traditional Chinese medicine/acupuncture, homeopathy, anthroposophic medicine, neural therapy, and herbal medicine) are currently covered by the mandatory basic health insurance when performed by a certified physician. Treatments by non-medical therapists are partially covered by a supplemental and optional health insurance. In this study, we investigated the frequency of CM use including the evolvement over time, the most popular methods, and the user profile.

**Methods:**

Data of the Swiss Health Surveys 2007 and 2012 were used. In 2007 and 2012, a population of 14,432 and 18,357, respectively, aged 15 years or older answered the written questionnaire. A set of questions queried about the frequency of use of various CM methods within the last 12 months before the survey. Proportions of usage and 95% confidence intervals were calculated for these methods and CM in general. Users and non-users of CM were compared using logistic regression models.

**Results:**

The most popular methods in 2012 were homeopathy, naturopathy, osteopathy, herbal medicine, and acupuncture. The average number of treatments within the 12 months preceding the survey ranged from 3 for homeopathy to 6 for acupuncture. 25.0% of the population at the age of 15 and older had used at least one CM method in the previous 12 months. People with a chronic illness or a poor self-perceived health status were more likely to use CM. Similar to other countries, women, people of middle age, and those with higher education were more likely to use CM. 59.9% of the adult population had a supplemental health insurance that partly covered CM treatments.

**Conclusions:**

Usage of CM in Switzerland remained unchanged between 2007 and 2012. The user profile in Switzerland was similar to other countries, such as Germany, United Kingdom, United States or Australia.

## Background

Since Eisenberg et al. published in 1993 that “the frequency of use of unconventional therapy in the United States is far higher than previously reported” [1], this topic has met growing interest in various parts of the world. The sum of unconventional therapies is often referred to as complementary and alternative medicine (CAM) or complementary medicine (CM). A research group within the European CAMbrella project defined CAM as follows: “CAM utilised by European citizens represents a variety of different medical systems and therapies based on the *knowledge*, *skills* and *practices* derived from *theories*, philosophies and *experiences used to maintain* and improve *health*, as well as to *prevent*, *diagnose*, relieve or *treat physical and mental illnesses*. CAM has been mainly used outside conventional health care, but in some countries certain treatments are being adopted or adapted by conventional health care.” (The words in italics are identical to the wording in the World Health Organisation’s definition, while the remaining wording was the result of a consensus process in the research group.) [2] Nevertheless, a comparison between surveys in various countries remains difficult due to the inclusion of different CM methods.

The utilisation of CM seems to have increased or remained stable over the last decades, e.g. in Australia from 20.3% in 1993 to 26.5% in 2004 [3]. In the United States, an increase in CM use from 33.8% in 1990 to 42.1% in 1997 was reported [4]. According to the National Health Interview Survey, CM use amounted to 32.3% in 2002, 35.5% in 2007 and 33.2% in 2012, without a significant difference between 2002 and 2012 [5]. In Taiwan, the use of traditional Chinese medicine (TCM) in children increased from 22.0% in 2005 to 22.5% in 2010, with an increasing trend of using herbal remedies [6].

A recent study estimated 145,000 medical and 160,000 non-medical practitioners in Europe [7]. Acupuncturists and homeopaths were most frequently represented. If and how the patients’ growing demand of CM was paralleled by an increase in the number of practitioners and how this correlated remains unknown.

Gender, age, education, and illness are predictors of CM utilisation. In many countries, e.g. Switzerland, Germany, Italy, Denmark, United Kingdom, Canada, United States and Australia, women, people of middle age and people with higher levels of education or higher income are more likely to use CM [8, 9, 3]. In Switzerland, suffering from migraine, arthritis, allergies or depression was associated with increased probability of CM usage [10]. In the United States, CM was mostly used for conditions such as back, neck or joint problems, anxiety, colds or headaches [11]. An increase in diversity of symptoms has been observed in acupuncture patients in the United Kingdom from 1988 to 2002. While the proportion of patients with musculo-skeletal disorders decreased from 47.3% to 38.1%, the proportion of patients with gynaecological and obstetric orders increased [12]. In France, homeopathic physicians had slightly more patients with joint diseases, anxiety-depressive and sleep disorders or dermatological diseases than physicians in general practice who prescribed only conventional medicines [13].

The aims of this study were (i) to determine the current usage of CM in Switzerland, (ii) to describe the development since the previous survey in 2007, and (iii) to investigate the usage of CM in certain groups of the population, e.g. pregnant women or chronically ill people. Our focus lay on CM methods, which are perceived as CM in Europe (especially Switzerland) and are provided by therapists or physicians, rather than dietary supplements, self-care or spiritual healing and prayer.

## Methods

### Data source

The Swiss Federal Statistical Office provided the anonymised data of the Swiss Health Surveys 2007 and 2012 [14]. The Swiss Health Survey is performed every 5 years in a sample representative of the Swiss resident population from the age of 15 on. It consists of a telephone (or face to face) interview followed by a written questionnaire, since not all questions can be asked on the telephone (due to length of the interview, complexity of some questions, possible need for consulting documents, intimacy of some questions). The survey includes questions about people's state of health, lifestyle, alcohol and drug abuse, physical exercise, health insurance and use of health services. In 2012, there were 21,597 oral interviews (53% participation quota) and 18,357 of the subsequent written questionnaires (88% of the respondents of the telephone or face to face interviews) [14]. The sample of 2007 has been described before [9].

In Switzerland, analysis of anonymous data does not require the approval of an ethics committee. This study was carried out in accordance with the Helsinki Declaration and Swiss laws and regulations.

### Variables

For the analysis in the present study, socio-demographic data (from the telephone interview) and all questions related to CM (from the written questionnaire) were included. In particular, the following questions were used (items and response options are shown in S1 Questionnaire):

- How is your health in general?
- Are you currently pregnant?
- Do you suffer from any chronic disease or health problem (persisting for at least 6 months)?
- How important is your health for you?
- Have you been to any physician within the last 12 months?
- How often have you been to one of the following specialists in the last 12 months: naturopath?
- How often have you used one of the following therapies in the last 12 months: acupuncture; traditional Chinese medicine; homeopathy; herbal medicine; shiatsu/foot reflexology; Indian medicine/ Ayurveda; osteopathy; other therapies, e.g. kinesiology, Feldenkrais method etc.?
- Do you have a supplemental health insurance for CM?

Persons who answered that they had seen a naturopath or used at least one CM therapy once were coded as CM users.

### Statistical analysis

IBM SPSS Statistics 21.0 (Armonk, NY, USA) including the complex samples module [15] was used for statistical analysis. The canton as stratum variable and weights of either the telephone or the written questionnaire were included, correspondingly. The numbers of respondents given in the tables correspond to the actual numbers in the survey (without weights).

Logistic regression models, which belong to the family of generalised linear models and are applied for binomial regression, were employed. For model 1, age group, gender, level of education were determined as categorical predictor variables, with usage of CM (1 = used at least once in the previous 12 months or 0 = never used in the previous 12 months) as the response variable.

For model 2, age group, gender, level of education and health-consciousness were chosen as categorical predictor variables, with having a supplemental health insurance for CM as the response variable.

Age was not used as a continuous variable, since it was not linear in the models. Predictor variables were chosen that were identified from previous studies to influence the use of CM (age, gender, educational level). Odds ratios (OR) and 95% confidence intervals (95% CI) were calculated from single factors of the logit function.

Two proportions were considered significantly different, when their 95% CI did not overlap, which is considered a conservative method compared to standard hypothesis testing [16].

## Results and Discussion

### Usage of CM in 2012

In Switzerland, the most popular CM methods of the adult population in 2012 were homeopathy, visits to naturopaths and osteopathy, followed by other therapies, herbal medicine, acupuncture, and shiatsu/foot reflexology (Table 1). The average number of treatments with any CM method in the previous year was approximately 8, ranging from 3 visits for homeopathy to 6 for acupuncture (Table 2). For all methods, the median number of visits was lower than the mean, indicating a skewed distribution. The literature provides little information on the number of visits that are typical for the course of a treatment with specific CM methods for comparison with our results. Homeopathic observational studies reported e.g. 1 to 5 consultations within on average 26.5 weeks for the treatment of atopic dermatitis [17] or approximately 8 consultations within 24 months for migraine [18]. Compared to clinical trials with acupuncture or osteopathy, the number of treatments in Table 2 was also similar or lower [19, 20].

**Table 1:** Usage of various methods of CM within 12 months.

	2012		2007	
Method	N (unweighted)	% (weighted)	N (unweighted)	% (weighted)
Any	5018	25.0 (24.2–25.8)	3458	24.0 (23.1–25.0)
Homeopathy	1662	8.2 (7.7–8.7)	893	6.4 (5.8–6.9)
Naturopath	1597	7.7 (7.2–8.2)	1185	7.7 (7.2–8.3)
Osteopathy	1459	6.8 (6.4–7.2)	838	5.4 (5.0–5.9)
Other methods^a^	1242	6.1 (5.7–6.6)	1113	7.8 (7.2–8.4)
Herbal medicine	1014	5.0 (4.6–5.4)	422	2.7 (2.4–3.1)
Acupuncture	1007	4.9 (4.5–5.3)	716	4.9 (4.5–5.4)
Shiatsu/foot reflexology	863	4.3 (4.0–4.7)	707	4.8 (4.4–5.3)
TCM	391	1.9 (1.7–2.2)	235	1.7 (1.5–2.0)
Ayurveda	202	0.9 (0.8–1.1)	141	1.0 (0.8–1.3)

Weighted percentages with 95% confidence intervals and unweighted numbers of respondents are presented (Swiss Health Survey 2007 and 2012).

^a^ In 2007, separate questions were asked for neural therapy, anthroposophic medicine, bioresonance therapy, and autogenic training or hypnosis. These users were added here to “other methods”.

**Table 2:** Average number of treatments with CM methods within 12 months (Swiss Health Survey 2012).

Method	Unweighted number of respondents	Mean (SD)	Median (Range)
Any	5018	7.73 (3.45)	4 (1–123)
Homeopathy	1662	2.96 (2.07)	2 (1–52)
Naturopath	1597	4.17 (2.42)	2 (1–40)
Osteopathy	1459	3.74 (2.43)	3 (1–40)
Other methods	1242	5.34 (2.80)	3 (1–92)
Herbal medicine	1014	3.22 (2.31)	2 (1–52)
Acupuncture	1007	6.19 (2.83)	4 (1–80)
Shiatsu/foot reflexology	863	4.65 (2.42)	3 (1–48)
Traditional Chinese medicine	391	4.96 (2.82)	3 (1–40)
Ayurveda	202	3.12 (2.05)	2 (1–40)

Investigation of the socio-demographic characteristics of the CM users revealed that people between 25 and 64 years were more likely to use a CM therapy than younger or older ones. Women as well as people with a higher education used CM to a higher degree than men and less educated persons, respectively (Table 3). This user profile is similar to the one found in the previous Swiss health survey [9] and in other countries [3].

**Table 3:** Logistic regression model: usage of CM in the last 12 months (Swiss Health Survey 2012).

			95% Confidence interval	
	Unweighted number of respondents	Odds ratio	Lower	Upper
**Age group**				
15–24	2983	0.769	0.665	0.890
25–44	6192	0.990	0.893	1.098
45–64	7539	1		
65 and above	4774	0.614	0.544	0.693
**Gender**				
Men	10225	1		
Women	11263	2.560	2.337	2.804
**Level of education**				
Compulsory school	3868	0.612	0.531	0.706
Upper secondary level	11568	1		
Tertiary level	6052	1.410	1.277	1.555

Additional analyses revealed that this utilisation profile was equal for all the individual methods with the exception of the use by age groups. Compared to the reference group of people between 45 and 64 years, homeopathy was applied to a higher extent by people between 15 and 24 years (OR = 1.489, 95% CI = 1.221–1.816), and shiatsu/foot reflexology as well as TCM were applied to a lower extent by people between 25 and 44 years (OR = 0.727, 95% CI 0.587–0.900 and 0.646, 95% CI 0.476–0.876, respectively).

In 2012, in Switzerland 5 CM methods were covered by the mandatory basic health insurance when performed by a certified physician, i.e., traditional Chinese medicine/acupuncture, homeopathy, anthroposophic medicine, neural therapy, and herbal medicine. However, 59.9% (95% CI 58.9%-60.9%) of the adult population had a supplemental insurance that covered part of the CM treatments. It is likely that many people decided to maintain their supplemental insurance, since it was announced by the Federal Department of Home Affairs in 2014 that coverage of the aforementioned 5 methods by the basic insurance was restricted until 2017, time limits were applied (e.g. 3 hours of acupuncture within 6 months), and other methods were not covered. Persons between 45 and 64 years, women, people with a higher education, and those reporting health-consciousness were more likely to have such a supplemental insurance compared to people of other age groups, men, persons with lower education, and without health-consciousness (Table 4).

**Table 4:** Logistic regression model: Holding a supplemental health insurance for CM (Swiss Health Survey 2012).

			95% Confidence interval	
	Unweighted number of respondents	Odds ratio	Lower	Upper
**Age group**				
15–24	2328	0.705	0.596	0.834
25–44	5192	0.783	0.705	0.871
45–64	6517	1		
65 and above	3984	0.768	0.686	0.860
**Gender**				
Men	8501	1		
Women	9520	1.670	1.529	1.824
**Level of education**				
Compulsory school	2685	0.791	0.683	0.915
Upper secondary level	5489	1		
Tertiary level	9847	1.120	1.017	1.234
**Health-conscious**				
No	2466	1		
Yes	15555	1.363	1.195	1.556

### Changes in use of CM between 2007 and 2012

In contrast to the analyses of the survey of 2007 [9], the present operationalization of CM use included visits to naturopaths. Furthermore, in 2007 bioresonance therapy, anthroposophic medicine, neural therapy, and autogenic training/hypnosis were inquired separately, while in 2012 these methods were subsumed under “other therapies”. To investigate any change between the two surveys, the values for 2007 were recalculated in order to fit the categories of 2012.

Table 1 compares the use of CM in 2007 and 2012. While overall usage did not change, an increase was evident in utilisation of homeopathy (primarily due to women’s use), osteopathy, and herbal medicine, whereas a decrease resulted for other methods. However, in 2007 separate questions had been asked for 4 more methods. These were retrospectively added to the group of “other methods”, which could have caused the higher percentage of users in 2007 compared to 2012.

For comparison: The percentage of people who saw any physician within 12 months was unchanged from 2007 (79.9%, 95% CI 79.1%-80.7%) to 2012 (78.4%, 95% CI 77.7%-79.1%).

Holding a supplemental health insurance for CM was not comparable to the survey from 2007 due to a different choice of answers in the questionnaire.

### Usage of CM by specific groups in 2012

We tried to determine the usage of CM by specific groups. When interpreting the answers, it must be considered that certain questions as e.g. about pregnancy were asked at the time point of the survey, while usage of CM was asked about within 12 months before the survey.

**Fig 1:**
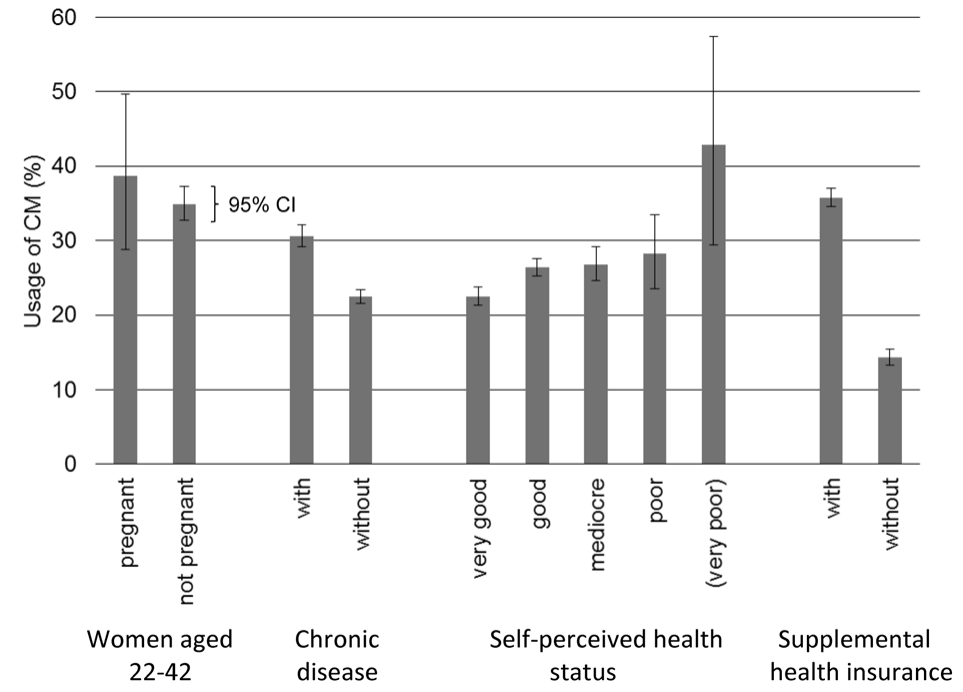
Usage of CM in various groups. Percentage and 95% confidence intervals (CIs) are shown. Characteristics were asked for at the time of the survey, while usage of CM was asked about within 12 months before the survey (Swiss Health Survey 2012). Groups with less than 30 answers are in parentheses.

Women who were pregnant at the time of the survey used as much CM as women of the same age (22 to 42 years) who were not pregnant (Fig 1). Homeopathy was used by 23.3 (95% CI 14.9%-34.6%) of pregnant women, i.e. almost twice as frequently as by nonpregnant women of the same age. For comparison, 26.7% of women in the United Kingdom reported using any form of CM during pregnancy. Herbal teas were most popular (17.7%), followed by homeopathy (14.4%) [21]. Moreover, in the United States, 36.7% of pregnant women and 27.8% of postpartum women reported using CM in the last 12 months compared with 40.7% of nonpregnant and non-postpartum women. No significant difference between pregnant and nonpregnant women was observed, while CM use by postpartum women was significantly lower [22].

People in Switzerland with a supplemental health insurance for CM used all CM methods except Ayurveda more commonly than people without a supplemental health insurance. Naturopaths were consulted 4 times as often, osteopathy, acupuncture, TCM, and other non-specified methods were employed more than 3 times as often as by people without a supplemental health insurance.

Additionally, our analysis revealed that people with a chronic disease or health problem used all CM methods except Ayurveda more commonly than people without a chronic health problem. Acupuncture and TCM were used almost twice as often. Similarly, an analysis of the data from 2007 had revealed that suffering from certain diseases correlated with increased CM [10]. Other countries have reported higher prevalence of CM users with specific chronic illnesses. E.g. in Singapore, 84% of people with chronic pain ever used CM compared to 76% in the general population. It was thus higher than in Western countries, and TCM, especially acupuncture, was most commonly used [23]. In a part of Norway, 62% and 73% of people with primary and secondary chronic headache had ever used CM for this condition [24]. People with type 2 diabetes and/or cardiovascular disease living in Victoria, Australia, used CM mostly along with conventional health care to maintain their health or assist in the management of their chronic condition. 22.1% of these patients never and 36.5% only sometimes discussed their CM use with medical doctors due to negative attitudes that they had experienced. Some patients received contradictory advice from CM practitioners and medical doctors, thus, better communication between these parties would be desirable [25]. 43.2% of patients with arthritis and 45.9% of patients with inflammatory bowel disease mostly from North America described themselves as current users of provider-based CM methods. More than 80% of these people used CM as a complement rather than as an alternative to conventional medicine. Additionally, seeing oneself as having a healthy lifestyle predicted CM use [26].

### Limitations

The sample in this survey was large (18,357 respondents) and representative of the Swiss population aged 15 and older living in a private household. Elderly people (living in e.g. nursing homes) and people with poor language skills in German, French or Italian may have been underrepresented. The answers were self-declared and may have been influenced by social desirability and recall bias. Since the questions about the usage of CM were part of the written questionnaire, respondents had the possibility to check the frequency of practitioner visits from their documents before answering, but it remains unknown if they did so. Some of the categories of CM methods in the questionnaire were unusual, e.g. the separation of acupuncture and TCM or the combination of shiatsu and foot reflexology. No definitions of the methods were provided.

## Conclusions

The usage of CM in Switzerland remained unchanged between 2007 and 2012. Homeopathy and osteopathy were the most popular CM methods with 8.2% and 6.8% of users, respectively. The user profile (CM users were more frequently female, of middle age, and with higher education) was similar to other countries. Patients’ preference for CM is substantial in Switzerland: Physicians should be aware that 30.5% of chronically ill people and 35.8% of people with an additional health insurance use CM.

## Supporting Information

S1 Questionnaire Excerpt of questions used from the questionnaire of the Swiss Health Survey 2012. (PDF)
