# Correction: Usage of Complementary Medicine in Switzerland: Results of the Swiss Health Survey 2012 and Development Since 2007

**DOI:** 10.1371/journal.pone.0144676

**Published:** 2015-12-09

**Authors:** Sabine D. Klein, Loredana Torchetti, Martin Frei-Erb, Ursula Wolf

There are errors in the column labeled “Unweighted number of respondents” in both Tables 3 and 4. Please view the correct Tables 3 and 4 here.

**Table 3:** Logistic regression model: usage of CM in the last 12 months (Swiss Health Survey 2012).

			95% Confidence interval	
	Unweighted number of respondents	Odds ratio	Lower	Upper
**Age group**				
15–24	2330	0.769	0.665	0.890
25–44	5216	0.990	0.893	1.098
45–64	6514	1		
65 and above	3932	0.614	0.544	0.693
**Gender**				
Men	8474	1		
Women	9518	2.560	2.337	2.804
**Level of education**				
Compulsory school	2660	0.612	0.531	0.706
Upper secondary level	9856	1		
Tertiary level	5476	1.410	1.277	1.555

**Table 4:** Logistic regression model: Holding a supplemental health insurance for CM (Swiss Health Survey 2012).

			95% Confidence interval	
	Unweighted number of respondents	Odds ratio	Lower	Upper
**Age group**				
15–24	1300	0.705	0.596	0.834
25–44	4564	0.783	0.705	0.871
45–64	6055	1		
65 and above	3611	0.768	0.686	0.860
**Gender**				
Men	7261	1		
Women	8269	1.670	1.529	1.824
**Level of education**				
Compulsory school	1898	0.791	0.683	0.915
Upper secondary level	8575	1		
Tertiary level	5057	1.120	1.017	1.234
**Health-conscious**				
No	1918	1		
Yes	13612	1.363	1.195	1.556
