# Supplementary material for: Usage of Complementary Medicine in Switzerland: Results of the Swiss Health Survey 2012 and Development Since 2007
Source: PLoS One. 2015 Oct 29;10(10):e0141985. doi: 10.1371/journal.pone.0141985 (PMC4626041; doi:10.1371/journal.pone.0141985)
Supplement: S1 Questionnaire — (PDF) [file pone.0141985.s001.pdf]

Supplementary material: Excerpt of questions used from the questionnaire of the Swiss Health Survey 2012

S.D. Klein, L. Torchetti, M. Frei-Erb, U. Wolf

The full questionnaire can be found in German, French and Italian on the website of the Swiss Federal Statistical Office:

http://www.bfs.admin.ch/bfs/portal/de/index/infothek/erhebungen__quellen/blank/blank/ess/03.html

The following questions from the telephone interview were used in our study:

10.00 How is your state of health in general? Is it…

- Very good
- Good
- Mediocre
- Poor
- Very poor
- Don't know
- No answer

11.00 Do you suffer from any disease or health problem that is chronic or ongoing?

This refers to diseases or health problems that have persisted for at least 6 months or will persist for approximately at least 6 months.

- Yes
- No
- Don't know
- No answer

22.00 Only for women between 15 and 49 years:

Are you currently pregnant?

- Yes
- No
- Don't know
- No answer

26.00 Have you been to any physician within the last 12 months (including specialists and gynaecologists, without dentists)?

- Yes
- No
- No answer

The following questions from the written questionnaire were used in our study:

1 How important is your health for you? Here you can see 3 different opinions. Please indicate, which one agrees best with your own opinion.

- I live without paying attention to possible consequences for my health.
- My lifestyle is influenced by thoughts about maintaining my health.
- Health considerations greatly determine how I live.

6 How often have you been to one of the following specialists in the last 12 months due to health related problems or control visits?

If you have never been to the respective persons, please enter each time the number "0".

- Naturopath.._ _ times

(Further specialists are not listed here, because they are not relevant to our study.)

7 How often have you used one of the following therapies in the last 12 months?

If you have never used one of the following therapies, please enter each time the number "0".

a) Acupuncture ...._ _ times

b) Traditional Chinese medicine (without acupuncture) ...._ _ times

c) Homeopathy ...._ _ times

d) Herbal medicine ...._ _ times

e) Shiatsu / Foot reflexology ...._ _ times

f) Indian medicine / Ayurveda ...._ _ times

g) Osteopathy ...._ _ times

h) Other therapies, e.g. kinesiology, Feldenkrais method, autogenic training

neural therapy, bioresonance therapy, anthroposophic medicine................._ _ times

27 Do you have a supplemental health insurance for complementary medicine?

- Yes

- No

- Don’t know
